# Apilimod Inhibits the Production of IL-12 and IL-23 and Reduces Dendritic Cell Infiltration in Psoriasis

**DOI:** 10.1371/journal.pone.0035069

**Published:** 2012-04-06

**Authors:** Yumiko Wada, Irma Cardinale, Artemis Khatcherian, John Chu, Aaron B. Kantor, Alice B. Gottlieb, Noriaki Tatsuta, Eric Jacobson, James Barsoum, James G. Krueger

**Affiliations:** 1 Synta Pharmaceuticals Corp, Lexington, Massachusetts, United States of America; 2 Laboratory for Investigative Dermatology, The Rockefeller University, New York, New York, United States of America; 3 Biomarker Discovery Sciences, Pharmaceutical Product Development Inc., Menlo Park, California, United States of America; 4 Department of Dermatology, Tufts Medical Center, Boston, Massachusetts, United States of America; Istituto Superiore di Sanità, Italy

## Abstract

Psoriasis is characterized by hyperplasia of the epidermis and infiltration of leukocytes into both the dermis and epidermis. IL-23, a key cytokine that induces T_H_17 cells, has been found to play a critical role in the pathogenesis of psoriasis. Apilimod is a small-molecule compound that selectively suppresses synthesis of IL-12 and IL-23. An open-label clinical study of oral administration of apilimod was conducted in patients with psoriasis. Substantial improvements in histology and clinical measurements were observed in patients receiving 70mg QD. The expression of IL-23p19 and IL-12/IL-23p40 in skin lesions was significantly reduced in this dose group, with a simultaneous increase in IL-10 observed. A decrease in the levels of T_H_1 and T_H_17 cytokines/chemokines in skin lesions followed these p19 and p40 changes. In parallel, a reduction in skin-infiltrating CD11c^+^ dendritic cells and CD3^+^ T cells was seen, with a greater decrease in the CD11c^+^ population. This was accompanied by increases in T and B cells, and decreases in neutrophils and eosinophils in the periphery. This study demonstrates the immunomodulatory activity of apilimod and provides clinical evidence supporting the inhibition of IL-12/IL-23 synthesis for the treatment of T_H_1- and T_H_17-mediated inflammatory diseases.

## Introduction

Psoriasis vulgaris is one of the most prevalent cell-mediated inflammatory diseases in humans [1] and serves as a model in which the activity and immune mechanisms of new therapeutics can be readily evaluated in affected tissues. Recent data from inflammatory skin models suggests that IL-23 and T_H_17 T cells, which produce IL-17 and IL-22, could be key inducers of epidermal hyperplasia and altered epidermal differentiation in psoriasis [2], [3]. This pathway is implicated by a marked increase in IL-23 synthesis [4] and T_H_17 T cells are found in psoriasis lesions [5], [6]. Genetic study has demonstrated the association of the IL-23/Th17 pathway with susceptibility to psoriasis [7]. A decrease in expression of p19 and p40 mRNAs (encoding IL-23) was observed in patients responding to some immune-modulating treatments [8], [9]. Clinically significant efficacy in the treatment of moderate to severe chronic plaque psoriasis was recently demonstrated by ustekinumab (CNTO-1275) and briakinumab (ABT-874), which both target the common p40 subunit of IL-12 and IL-23, confirming the major role of IL-12 and IL-23 in the pathophysiology of the disease [10], [11], [12], [13], [14]. Another newly recognized feature of psoriasis is that skin lesions are highly infiltrated by CD11c^+^ dendritic cells termed TIP-DCs (TNF- and iNOS-producing DCs), which also synthesize IL-20 and IL-23 in skin lesions [4], [15], [16]. Hence psoriasis brings together inflammatory pathways driven by CD11c^+^ DCs, T_H_1, and T_H_17 T cells, but in the context of an accessible human organ in which effective suppression of inflammation can fully reverse disease-defining pathology and restore normal cell growth and gene expression [17].

Successful clinical trials with antibodies directed against IL-12/IL-23 support the approach of modulating inflammation in psoriasis or other T cell mediated diseases by selectively blocking production of IL-12 and IL-23. Although antibodies can provide medical benefit, an orally available small-molecule IL-12/IL-23 inhibitor is also highly desirable. Apilimod (formerly STA-5326) is a small molecule that was developed from a novel triazine derivative identified through high-throughput IL-12 inhibitor screening [18]. Apilimod effectively suppresses synthesis of IL-12 and IL-23 in myeloid leukocytes and oral administration of apilimod led to a suppression of the T_H_1 but not T_H_2 immune response in mice [18]. *In vivo* studies demonstrated that oral administration of apilimod markedly reduced inflammatory histopathologic changes. A striking decrease in IFN-γ production was observed in *ex vivo* culture of cells harvested from animals treated with apilimod, indicating a down-regulation of the T_H_1 response by this compound.

In this study, patients with stable psoriasis vulgaris skin plaques were treated orally with a range of apilimod doses. Skin biopsies and whole blood were collected throughout a 12-week treatment course, and extensively analyzed by immunohistochemistry, RT-PCR, cytometry, and cytokine production levels in *ex vivo* cell culture, to measure inhibition of p40 cytokines and downstream products in the local site of inflammation as well as in the periphery. Our results establish that apilimod not only suppresses synthesis of IL-12, IL-23, and multiple downstream cytokines in the lesional skin, but also concomitantly increases synthesis of the anti-inflammatory cytokine IL-10. This study also presents an overall view of the action of this IL-12/IL-23 blocker, and provides additional evidence for critical links between IL-23 synthesis, production of IL-17 at elevated levels in psoriasis, and resulting histopathological alterations in the skin.

## Results

### Apilimod Treatment of Human whole Blood *In Vitro* Leads to a Concurrent Decrease of IL-12 and Increase of IL-10 and GM-CSF

**Figure 1:**
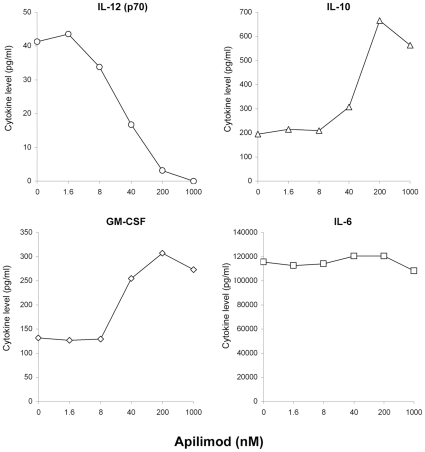
*In vitro* effect of apilimod on IL-12p70, IL-10, GM-CSF, and IL-6 in human whole blood cells. Human whole blood from a normal volunteer was stimulated with 0.1% SAC in the presence of different concentrations of apilimod. Supernatants were tested for IL-12p70 (circles), IL-10 (triangles), GM-CSF (diamonds) and IL-6 (squares). Results are representative of one of three individual experiments with whole blood from different volunteers each time.

It was previously reported that apilimod treatment inhibited IL-12 production in human PBMCs, monocytes, monocyte-derived dendritic cells, and the human monocytic cell line THP-1 with IC_50_ values below 20nM, while not significantly suppressing the production of other cytokines [18]. The selectivity of the compound was further evaluated using SAC-stimulated human whole blood. In this assay IL-12 production was consistently inhibited by apilimod with the IC_50_ ranging from 20 to 200nM (Fig. 1). Interestingly, IL-10 and GM-CSF production was reproducibly enhanced by the compound (Fig. 1). The increase of IL-10 and GM-CSF was dose-dependent and reached greater than 2-fold at drug concentrations above 200 nM. IL-6 production was neglibly affected in this assay.

### Decreased IL-12 and Increased IL-10 and GM-CSF in *Ex Vivo* Stimulated whole Blood Cells Drawn 2h Post Oral Administration of Apilimod

**Figure 2:**
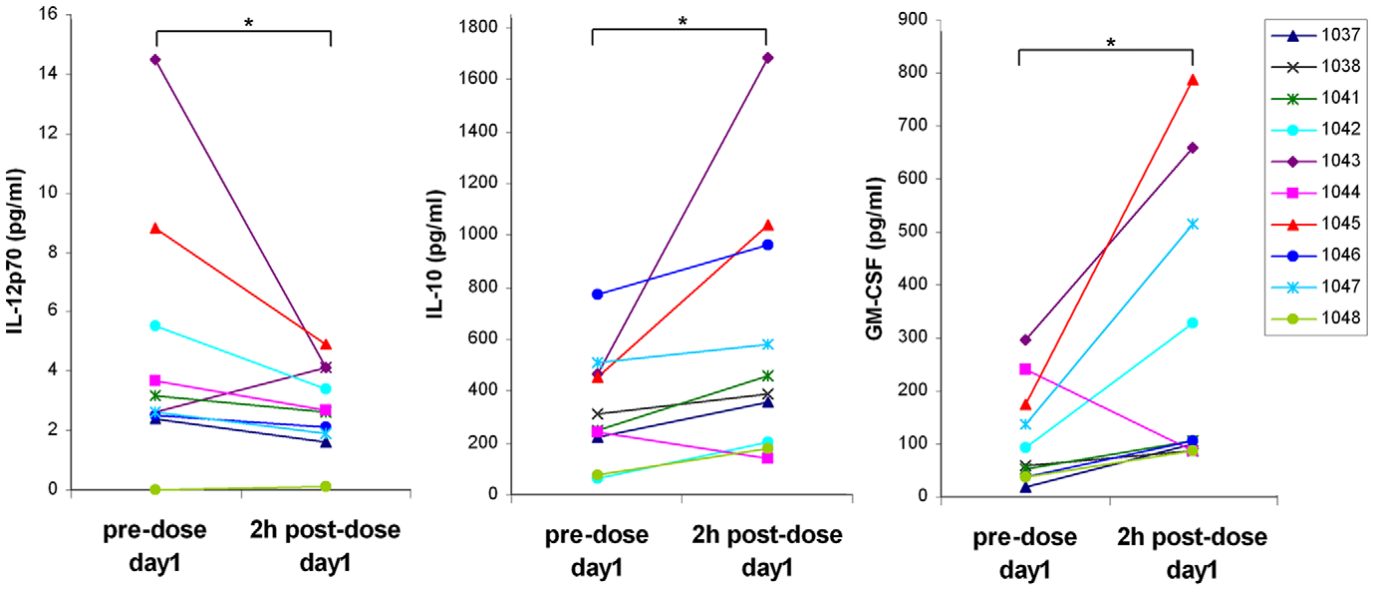
*Ex vivo* stimulation of whole blood drawn 2h post 70mg dose as compared to the pre-dose in cytokine production. Whole blood drawn pre-dose and 2h post 70mg dose (*n*  =  10) were stimulated with 0.1% SAC within 24h of the draw, and the supernatants were analyzed for IL-12p70 (a), IL-10 (b), and GM-CSF (c). *, *p* ≤0.05; statistically significant differences between pre- and 2h post-dose.

Apilimod was tested in a Phase 2a multi-center, open-label clinical trial in psoriasis patients in which the focus was a variety of biomarker-based measures of biological response. Patients with moderate to severe chronic plaque psoriasis received apilimod at doses of 21mg BID, 35mg QD, 35mg BID, or 70mg QD orally for 12 weeks. First, to confirm that the potency and selectivity of apilimod was similar to the *in vitro* results above, whole blood was collected pre- and 2 h post-dose to measure cytokine production in response to *ex vivo* SAC stimulation. The 2 h time point represents the approximate time at which the maximum apilimod concentration in plasma was observed (mean ± SD plasma levels at 2 h post-dose were 41 ± 41nM (*n*  =  16), 122 ± 82nM (*n*  =  16), 115 ± 60nM (*n*  =  14), and 265 ± 183nM (*n*  =  15) at the doses of 21mg BID, 35mg QD, 35mg BID, and 70mg QD, respectively). Figure 2 shows the changes in cytokine production levels in the *ex vivo* culture with the whole blood containing orally absorbed apilimod from the patients receiving the highest dose of 70mg. IL-12 production was decreased at 2 h post-dose compared to the pre-dose samples in several patients in the dose group (median change of -27%), while GM-CSF and IL-10 were concurrently increased (median change of +147% and +71%, respectively). A decrease in IL-12 compared to the corresponding pre-dose whole blood was also consistently observed in the other dose groups (data not shown). GM-CSF and IL-10 were also increased in the 35 mg apilimod cohort (both QD and BID), but not 21 mg. This is in agreement with the *in vitro* increases of these cytokines at a relatively high drug concentration compared to the concentration which caused a reduction in IL-12. The changes in cytokine production were not correlated with patient response, indicating that this *ex vivo* assay of peripheral blood at the apilimod C_max_ is not sufficient to predict clinical response.

### Clinical Response to Apilimod Treatment

**Figure 3:**
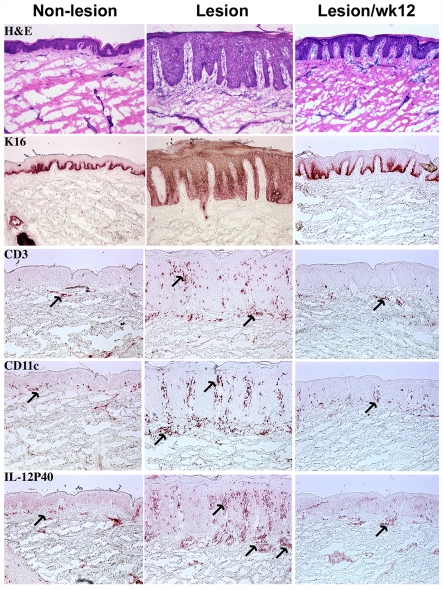
Histological improvement by apilimod treatment. Histology and immunohistochemistry of one patient (1046) showing improved histology and clinical measures (58%reduction in PASI score) at week 12 in the 70mg QD apilimod treated group. Skin biopsies from non-lesions (left) and lesions (middle) at baseline and lesion at week 12 (right) were stained with H&E, K16, anti-CD3 Ab, anti-CD11c Ab, or anti- IL-12p40 Ab. Cells staining positive for CD3, CD11c and IL-12p40 are indicated (arrows).

The primary efficacy endpoint of this biomarker study was the proportion of patients with an improvement in the histological assessment of skin biopsies. Frozen skin biopsies from non-lesion skin and psoriatic lesions were analyzed for routine histopathology, expression of keratin 16 (K16) and IL-12/IL-23p40, and numbers of CD3^+^ T and CD11c^+^ dendritic cells. Given the typical histopathology of baseline psoriasis, the grading system for the histological assessment of epidermal hyperplasia is: (1) not improved; (2) good improvement (reduction in hyperplasia and normalized differentiation, but most suprabasal keratinocytes still express K16); or (3) excellent improvement (reduction in hyperplasia, normalized differentiation, absent K16 expression or small foci of residual keratin expression). Patients with good or excellent improvement at week 12 were considered responders. Any patient missing data at week 12 was treated as a non-responder. Figure 3 shows a representative example of a responder who achieved excellent improvement. Overall clinical responses based on histological assessment of skin biopsies and other clinical measures in the four dose groups are shown in Table 1. There was a clear trend towards better clinical outcomes at the dose of 70 mg QD with half of patients showing significant histological improvement at week 12. Comparisons between dose groups in the clinical measures of PASI (Psoriasis Area and Severity Index) and sPGA (static Physician’s Global Assessment) also favored the 70mg QD cohort. Similar to the histological improvement, around half of patients achieved a 50% improvement in their PASI scores and showed a 2-point or greater improvement in PGA from week 0 to week 12 in this dose group (Table 1). Overall, 13 of 15 total patients with less than 30% reduction in PASI score at week 12 were defined as non-responders in histological assessment, and 9 patients with at least a 70% reduction in PASI score were all histological responders, indicating a close correlation between the histological assessment and the clinical measures.

**Table 1:** Clinical response based on assessment of skin biopsies and PASI and PGA.

	21 mg BIDn = 17n (%)	35 mg QDn = 17n (%)	35 mg BIDn = 16n (%)	70 mg QDn = 15n (%)
Histological Improvement	3 (18)	4 (24)	3 (19)	7 (47)
PGA Clear/Almost Clear	1 (6)	1 (6)	1 (6)	2 (13)
PASI 75	0	0	1 (6)	2 (13)
Mean Improvement in PASI	15%	29%	40%	46%
PASI 50	3 (18)	5 (29)	8 (50)	7 (47)
2 pt Improvement in PGA	3 (18)	4 (24)	5 (31)	8 (53)

### Infiltrating T Cells and Dendritic Cells Post-apilimod Treatment

**Figure 4:**
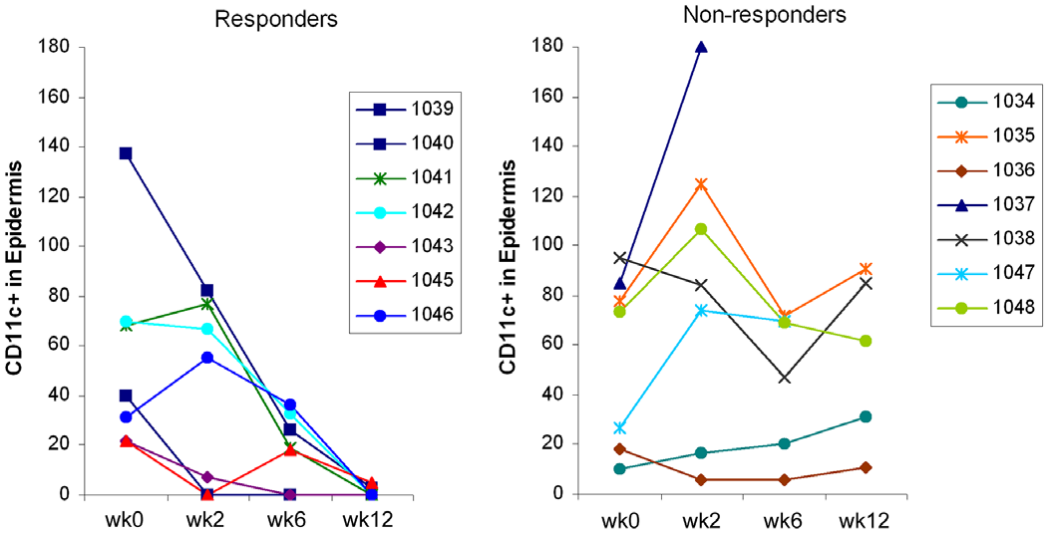
Changes in epidermal CD11c^+^ cells in responders and non-responders in 70mg QD apilimod cohort. Results shown are the number of epidermal CD11c^+^ cells in individual patients categorized as either responders (left, *n*  =  7) or non-responders (right, *n*  =  7) in the 70mg QD cohort at baseline (week 0), week 2, 6, and 12.

Immunohistochemisty of skin lesions demonstrated that the histological improvement was paralleled by progressive decreases in the number of infiltrating CD3^+^ (a marker of T cells) and CD11c^+^ (a marker of dendritic cells) cells (Fig. 3, Table 2). The number of CD3^+^ cells in both the epidermis and dermis was decreased at week 12 from baseline in histological responders (mean change ± SD: −46 ± 47% and −38 ± 49%, respectively), while no change was seen in non-responders (+5 ± 78% and +10 ± 69%, respectively). A dramatic decrease was seen in the number of CD11c^+^ cells in the responders (−78 ± 36% in epidermis and −52 ± 39% in dermis), while the decrease in non-responders was not significant (−21 ± 69% in epidermis and −2 ± 59% in dermis). The differences between responders and non-responders at week 12 were statistically significant for CD3^+^ cells and CD11c^+^ cells in both the epidermis and dermis (Table 2). Among the dose groups, the decreases in the infiltrating cells was most prominent in the 70mg QD cohort (*n*  =  12) for all of dermal CD3^+^ (−29%), epidermal CD3^+^ (−31%), dermal CD11c^+^ (−29%), and epidermal CD11c^+^ (−43%) cells at week 12. Within the 70mg QD dose group, the largest decrease was observed in epidermal CD11c^+^ cells with almost complete clearance in all responders (−96 ± 8%) (Fig. 4).

**Table 2:** Mean skin-infiltrating T cell and dendritic cell numbers.

Population^1^			wk0	wk2	wk6	wk12
Epidermal CD3^+^ cell	Mean cell number	non-responder	74	79	68	65
		responder	68	63	44	25
	p value between group		0.631	0.267	0.127	0.001
Dermal CD3^+^ cell	Mean cell number	non-responder	112	112	95	109
		responder	123	115	73	64
	p value between group		0.464	0.889	0.177	0.008
Epidermal CD11c^+^ cell	Mean cell number	non-responder	47	44	38	32
		responder	40	41	20	8
	p value between group		0.555	0.800	0.039	0.002
Dermal CD11c^+^ cell	Mean cell number	non-responder	142	141	127	118
		responder	140	154	93	56
	p value between group		0.929	0.623	0.158	0.001

1 Mean cell numbers of epidermal CD3^+^ (T cell) cells, dermal CD3^+^ cells, epidermal CD11c^+^ (dendritic cell) cells, and dermal CD11c^+^ cells per low-power field during treatment, with patients classified by response. There were 17 responders and 30 non-responders in the analysis.

### Gene Expression in Lesions Post-apilimod Treatment

**Figure 5:**
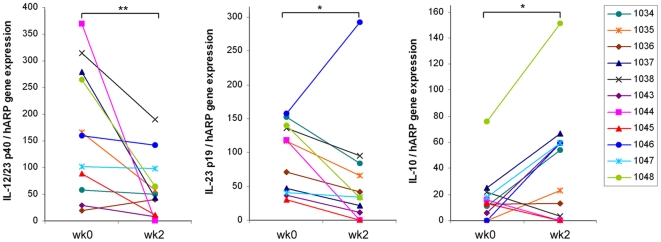
Changes in the expression levels of IL-12/IL-23p40, IL-23p19, and IL-10 at week 2 in 70mg QD apilimod cohort. RNA was prepared from biopsies obtained from the psoriatic skin lesions at baseline (week 0) and week 2, and RT-PCR was performed for IL-12/IL-23p40 (a), IL-23p19 (b), and IL-10 (c). The expression levels were normalized to house keeping gene, hARP. Results shown are the expression levels of individuals in 70mg QD cohort (*n*  =  11, 3 histological responders, 8 histological non-responders). *, *p* ≤0.05; **, *p* ≤0.01; statistically significant differences between baseline and week 2.

To determine the *in vivo* effects of apilimod on the expression of the target genes IL-12 and IL-23, as well as their downstream targets at the local site of inflammation, we conducted RT-PCR on skin biopsies collected before and after treatment. The level of gene expression in each biopsy was normalized to human acidic ribosomal phosphoprotein PO (hARP). This protein, whose mRNA level is stable regardless of treatment, was used to confirm the quality of the samples [19]. The level of IL-12/IL-23p40 and IL-23p19 expression at baseline was remarkably higher in psoriatic skin lesions (median normalized gene expression of 51.3 and 38.0, respectively, *n*  =  54) compared to corresponding normal skin (0 and 7.9, respectively). Significant decreases in p40 and p19 at week 2 from baseline were demonstrated in the 70mg QD group with median percent changes of −65% and −45%, respectively (Fig. 5). In contrast, IL-10 was increased with a +98% median percent change relative to the baseline (*n*  =  9) (Fig. 5). Two patients (1035 and 1046) excluded in the analysis of the percent change in IL-10 (as the baseline value was 0) also showed a dramatic increase in IL-10 (23 and 59 from 0). The IL-10 decrease seen in patient 1045 was accompanied by a decrease in epidermal CD11c^+^ cells at week 2, suggesting that decreased IL-10 was likely due to the loss of IL-10 producing cells in the epidermis, still consistent with increased IL-10 expression by apilimod treatment when no change in the infiltrating cell population is apparent.

**Figure 6:**
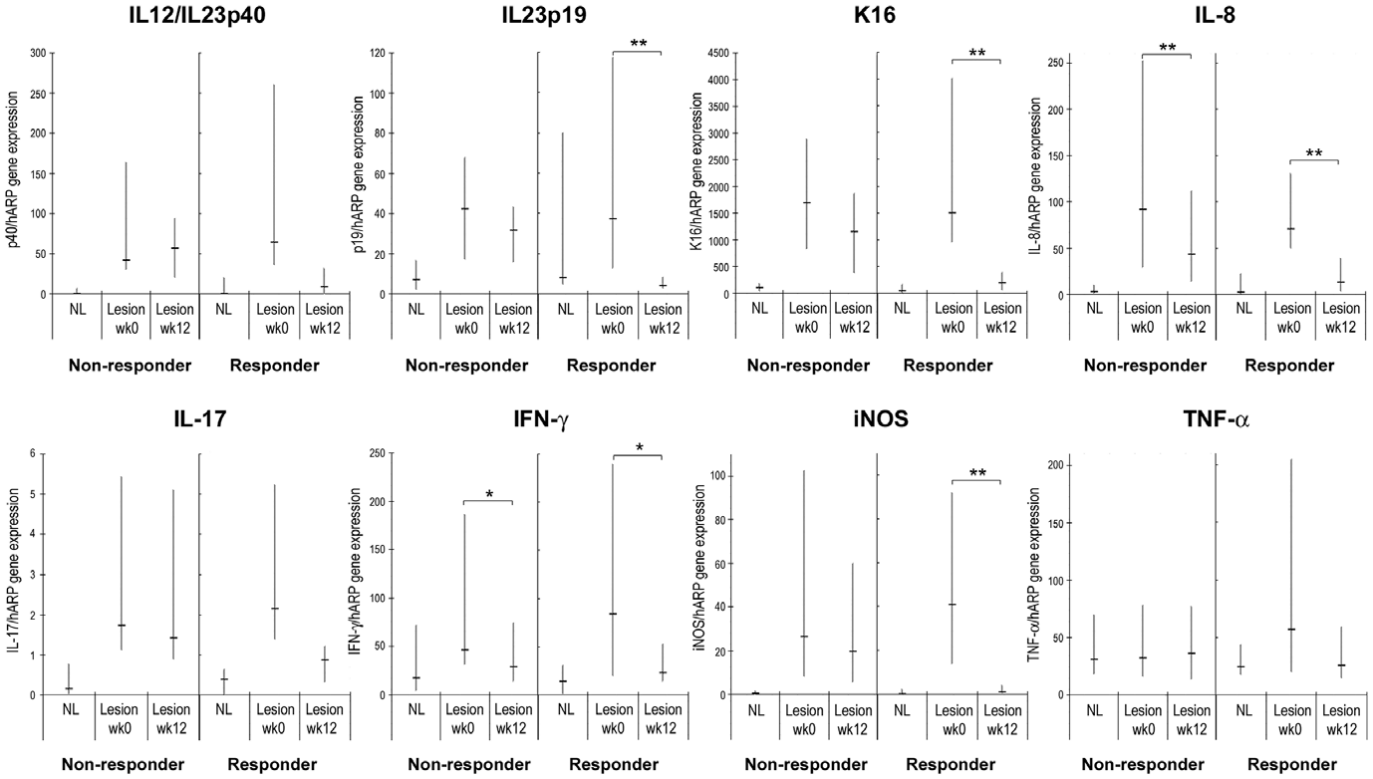
Changes in the expression levels of T_H_1, T_H_17, and other inflammatory genes at week 12 in responders and non-responders. RT-PCR was performed with biopsies obtained from non-lesion skin and psoriatic lesions. Results shown are the percentile range (25^th^–75^th^%, medians indicated) of histological responders (*n*  =  11) and non-responders (*n*  =  27) for IL-12/IL-23p40, IL-23p19, K16, IL-8, IL-17, IFN-γ, iNOS and TNF-α. There were 26 non-responders for IL-17, and 10 responders and 23 non-responders for IL-10 in the analysis of the change due to 0 value at baseline. *, *p* < 0.05; **, *p* <0.01; statistically significant differences between before (week 0) and after (week 12) apilimod treatment in responder and non-responder groups.

At week 12, the gene expression of IL-12/IL-23p40 and IL-23p19 was further reduced from baseline in the 70mg QD group (median changes of −74% and −81%, respectively, *n*  =  7). IL-10 gene expression remained higher than the baseline in the dose group (+141%, *n*  =  5). As predicted from the biological effects of IL-12/IL-23, one would expect inhibition of the downstream T cell and inflammatory pathways affected by IL12/IL-23 follows inhibition of these cytokines. Consistent with this view, a marked reduction in the gene expression of K16, iNOS, IL-8, IL-17, TNF-α, and IFN-γ was observed at week 12 (median changes of −51%, −58%, −61%, −23%, −43%, and −50%, respectively, *n*  =  7) while only small and inconsistent decreases were seen at week 2. Moreover, a nearly complete reduction in K16 and T_H_1/T_H_17 cytokines and chemokines was demonstrated in most of histological responders with the median gene expressions at week 12 comparable to the levels of non-lesional skin (Fig. 6). In contrast, no significant changes were seen in non-responders.

### Effect of Apilimod Treatment on Peripheral Cell Profile

**Figure 7:**
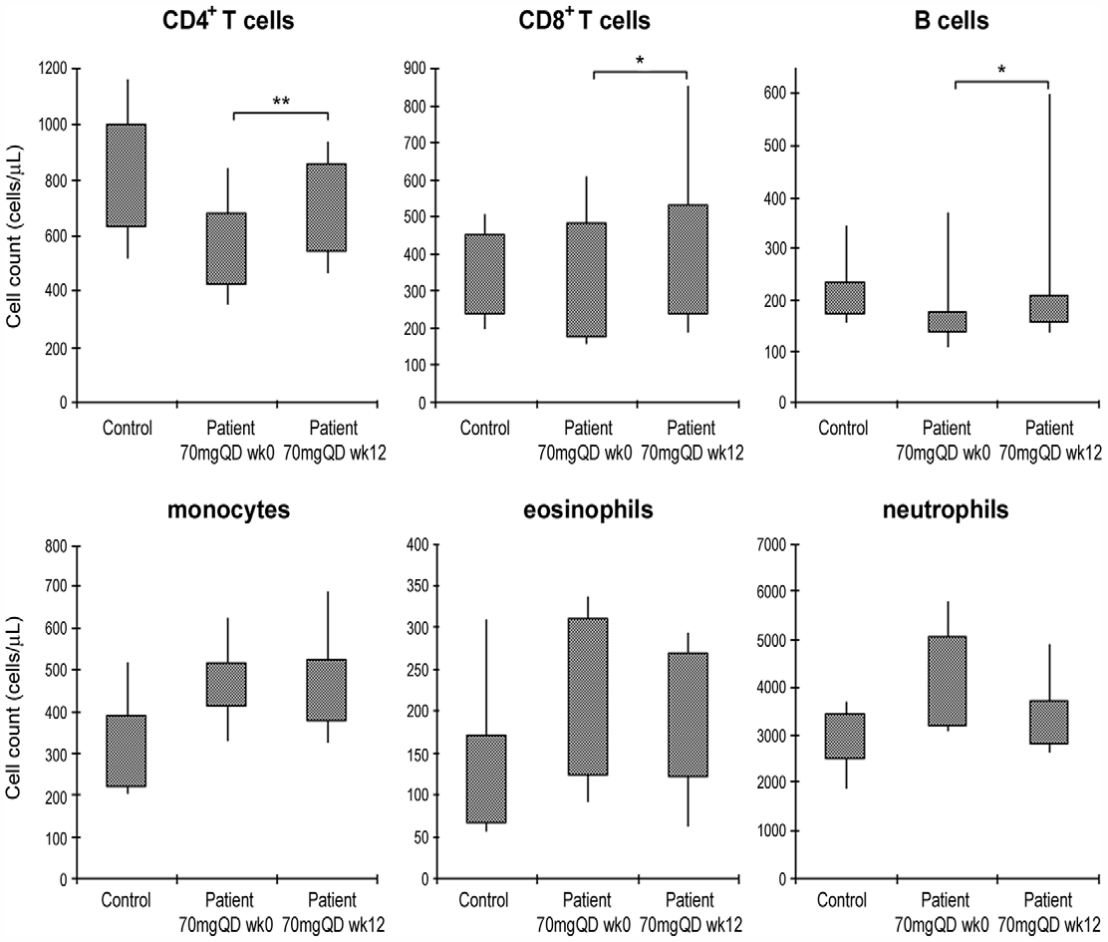
Cellular phenotype in normal controls and psoriasis patients before and after 70mg QD treatment. Whole blood cells from normal controls and psoriasis patients were analyzed by cytometry for the cellular phenotype. Results shown are the percentile ranges based on cell counts (cells/µL) of peripheral CD4^+^ T cells, CD8^+^ T cells, B cells, monocytes, eosinophils and neutrophils from normal controls (*n*  =  16) and psoriasis patients in 70mg QD cohort (*n*  =  12) at baseline (week 0) and week 12. Plot: bottom line, 10^th^%; bottom box, 25^th^%; top box, 75^th^%; top line, 90^th^%. *, *p* < 0.05; **, *p* <0.01; statistically significant differences between before (week 0) and after (week 12) 70mg QD apilimod treatment in psoriasis patients.

It is known that peripheral immune cells are recruited to sites of local inflammation [20], [21], [22]. To determine the effect of apilimod on the trafficking of immune cells, whole blood samples from patients were analyzed for immune cell profiling side-by-side with samples from normal controls. Neutrophil, eosinophil, and monocyte counts (mean ± SD cells/µL) at baseline were significantly higher in psoriasis patients than in normal controls (4476 ± 1699 vs. 3066 ± 947; *p*  =  0.001, 231 ± 152 vs. 138 ± 102; *p*  =  0.008, 420 ± 128 vs. 316 ± 123; *p*  =  0.004, *n*  =  60 and 16, respectively). In contrast, CD4^+^ T cell counts were slightly lower in psoriasis patients than in controls (742 ± 276 vs. 843 ± 283; *p*  =  0.125). There was no significant difference in CD8^+^ T and B cell counts between psoriasis patients and normal controls (354 ± 168 vs. 367 ± 135; *p*  =  0.679 and 220 ± 138 vs. 225 ± 78; *p*  =  0.200, respectively). After 12 weeks of treatment with apilimod, neutrophil and eosinophil counts were decreased in the 70mg QD cohort, while the counts of CD4^+^ T, CD8^+^ T, and B cells were significantly increased (Fig. 7). The increase of CD4^+^ T, CD8^+^ T, and B cells is more pronounced when expressed as a percentage of whole blood cells (mean change ± SD: +33 ± 35%; *p*  =  0.002, +34 ± 45%; *p*  =  0.039, and +42 ± 41%; *p*  =  0.0005, respectively). These results indicate that apilimod treatment resulted in a shift toward the normal state. There was no significant change in the monocyte counts (Fig. 7) or percentage (+4 ± 21%, *p*  =  0.622) after apilimod treatment. Similar increases in peripheral CD4^+^ T, CD8^+^ T, and B cells, and a decrease in neutrophils after treatment with apilimod were also observed in histological responders (mean change ± SD in the percentage: +34 ± 58%, +33 ± 44%, +41 ± 53%, and −6 ± 13%, *n*  =  15), although the difference from non-responders (+13 ± 24%, +15 ± 28%, +25 ± 38%, and −1 ± 9%, *n*  =  28) did not achieve statistical significance in any cellular subset.

## Discussion

Apilimod is an orally available small molecule that selectively and potently inhibits IL-12 and IL-23 production. The IL-12 p35 and p40 promoter driven luciferase assay indicated that the compound inhibits transcription of both p35 and p40 genes [18]. Investigation of regulatory factors revealed that nuclear accumulation of c-Rel, but not other NF-κB family member p65 or p50, was impaired by apilimod (Y. Wada, unpublished data). It was recently shown that c-Rel specifically regulates expression of IL-12p35, IL-12/IL-23p40, and IL-23p19 [23], [24], [25], [26]. The findings adequately explain the selective inhibition of these genes by apilimod. Upregulated expression of c-Rel has been shown in DCs and other myeloid cells [26], further supporting the targeted activity of apilimod in these populations.

An elevation of IL-12/IL-23p40 mRNA and protein levels as well as IL-23p19 mRNA has been reported in psoriatic skin lesions [4], [27], [28]. Here, we show a consistent decrease in p40 and p19 mRNA in psoriatic lesions as early as week 2 following the initiation of apilimod treatment in the highest dose group. There was no notable decrease in infiltrating cells at this early time point, suggesting that the mRNA changes are due to direct effects of apilimod on cytokine expression in cells, and not a result of changes in the cell number itself. At week 12, marked reductions in the gene expression including T_H_1/T_H_17 cytokines and chemokines paralleled the clearance of infiltrating cells in the responders, suggesting that mRNA reduction is partially due to the loss of cytokine-producing cells. Since CD3^+^ T cells were not totally cleared, the dramatic reductions in the levels of T_H_1/T_H_17 genes are likely due to a selective clearance of T_H_1/T_H_17 cells from the lesions and/or a marked suppression of the gene expression as a consequence of the clearance of IL-12/IL-23 producing CD11c^+^ cells. Importantly, clinical response correlated with the suppression of T_H_1/T_H_17 and downstream genes at week 12, validating the relevance of targeting this pathway for disease improvement.

In this study, a near-complete clearance of epidermal CD11c^+^ cells from lesions was observed in the responders in the 70mg QD group at week 12. A statistically significant reduction of CD3^+^ T cells in the responder group was also seen, however the reduction in CD3^+^ T cell numbers was less than the reduction of CD11c^+^ cells in both the epidermis and dermis. It is noteworthy that psoriasis clinical studies of ustekinumab [29], alefacept (CD2 binding portion LFA-3)[8], and etanercept (TNF-receptor fusion protein)[9] all showed greater decreases in CD3^+^ T cells compared to CD11c^+^ cells. A comparative reduction in CD3^+^ T cells and CD11c^+^ cells was achieved in a clinical study of efalizumab (anti-CD11a) [15]. The preferential reduction in CD11c^+^ cells by apilimod seen in the responders may be a consequence of its primary effect on this population. Because a good correlation between hyperplasia improvement and CD11c^+^ cell numbers in skin lesions has been consistently observed [8], [9], [15], clearance of CD11c^+^ cells is expected to result in faster resolution of psoriatic lesions and may help achieve better clinical outcome in combination with other agents that primarily target T cells.

Interestingly, we found that baseline levels of TNF-α and IFN-γ mRNA were higher in responders than non-responders (Fig. 6). The difference in baseline TNF-α was also pronounced in 70mg QD group (mean ± SD: 198 ± 108, *n*  =  3 vs. 59 ± 42, *n*  =  7). A similar correlation of TNF-α mRNA at baseline with response to treatment was seen in the ustekinumab trial [29]. The consistent observation of a correlation in two independent studies suggests that when inhibiting IL-12/IL-23, baseline TNF-α level may represent a potential molecular predictor of response.

IL-10 was reproducibly increased in *in vitro* culture following apilimod treatment as well as in lesions and *ex vivo* culture of whole blood cells following oral administration. No similar increase was observed in the clinical study of ustekinumab [29], suggesting that IL-10 modulation is due to a direct effect of apilimod, not secondary to the inhibition of IL-12/IL-23. A significant increase in the expression of GM-CSF by apilimod was also seen *in vitro* and *ex vivo*. Increased IL-10 and GM-CSF levels have been reported to be beneficial in the treatment of inflammatory disease [30], [31]. Clinical efficacy with IL-10 treatment in psoriasis patients was reported, with immunohistologic improvement [32], [33], as well as lowered incidence of relapse and prolonged disease-free interval [34]. Thus, the enhanced IL-10 effects of apilimod are expected to add benefit to clinical outcome.

We investigated multiple dose groups in this study, and identified the highest responses in the 70mg QD dose cohort for both histological and clinical improvements. Despite the fact that the range of apilimod doses in the four groups was narrow, the difference between 70mg QD group and 21mg BID group was apparent in gene expression of IL-23p19 in skin lesions at week 12 (−81% vs. +20%, *p*  =  0.192). The two doses are more distinct in the C_max_ than the total AUC and C_trough_. In addition, the 70mg QD group demonstrated a better clinical response than the 35mg BID group despite the similar AUC_0–24h_ in the 70mg QD and the 35mg BID cohorts, suggesting that a sufficiently high drug plasma concentration for a short duration rather than the total AUC determines efficacy. As the IL-12/IL-23 producing CD11c^+^ cells, the target of apilimod, are localized in the inflamed skin [4], the superior response of 70mg QD group implies that a higher drug level may have been achieved and maintained in the skin layers at this dose compared to others.

Ustekinumab has demonstrated great clinical success and has been approved for psoriasis treatment. How does apilimod differ from this antibody in terms of clinical response based on PASI 75? In a dose-escalating study in which patients received an i.v. infusion of 0.1, 0.3, 1, or 5 mg/kg of ustekinumab, significant improvement was observed at the two highest dose levels [29]. However, the mean PASI reduction shown by a sub-optimal 0.3mg/kg dose of the antibody was approximately 50% at week 12 [29], similar to the mean PASI reduction achieved by the 70mg QD apilimod dose. In addition, both ustekinumab and apilimod demonstrated reduction of skin infiltrated CD11c^+^ or CD3^+^ cells in responders with the reduction appearing slower for apilimod (6 weeks vs. 2 weeks for ustekinumab) [29]. It is reasonable to suggest, therefore, that the lower therapeutic activity of apilimod is likely due to insufficient drug levels to achieve an optimal clinical response. In this regard apilimod dosing is limited to 70 mg BID as CNS-related adverse events (headache, flushing, hypoesthesia, dizziness and paresthesia) were observed at a 105 mg BID dose level in a previous Phase I study (Y. Wada, unpublished data).

This report does however highlight the therapeutic promise of a small molecule IL-12/IL-23 inhibitor. The clear dose-response reduction in production/expression of IL-12/IL-23, number of infiltrating immune cells, as well as the clinical measures of PASI and PGA, implies that removing the dose-limiting toxicity with a derivative of apilimod and increasing the dose might produce improved clinical effects similar to the IL-12/IL-23 antibody. Moreover, the pronounced effects on dendritic cells over T cells and on IL-10 production/expression are unique to apilimod and may be biologically advantageous. In general, an orally available small molecule provides superior convenience and cost effectiveness for application, particularly in a chronic disease state such as psoriasis. In light of these potential advantages over an IL-12/IL-23 antibody such as ustekinumab, several apilimod derivatives with improved safety and pharmacokinetics are currently under investigation.

An important consideration of our findings relates to the prolonged therapeutic targeting of this pathway. Studies in mice deficient in c-Rel and IL-12 demonstrated that they are defective in T_H_1 [35], and have increased risk of infections for which T_H_1 and IL-12 are protective such as *Leishmania major* [36], [37], raising a question for risk of opportunistic infections by suppressing T_H_1 response. However, no evidence of immune-suppression was noted in comprehensive immune-toxicology evaluations of ustekinumab [38], supporting potential of long-term treatment with IL-12/IL-23 modulators.

In summary, here we have demonstrated the immunomodulatory activity of apilimod through extensive biomarker studies conducted in patients with moderate-to-severe psoriasis, by analyzing both local skin and peripheral blood samples using multiple methods in comparison with normal controls. The results provide an overall view of the action of apilimod. This novel agent suppressed the expression of IL-12/IL-23 in skin lesions and simultaneously enhanced IL-10. The expression of T_H_1 and T_H_17 cytokines/chemokines was reduced, accompanied by clearance of CD11c^+^ dendritic cells and CD3^+^ T cells from the skin lesions. Blocking the recruitment of immune cells to the skin resulted in an increase of their populations, and a decrease of granulocytes, in the periphery. This study clearly demonstrates the activity of the small molecule apilimod in psoriasis patients and further supports the critical role of the IL-23/T_H_17 pathway in the etiology of this disease.

## Materials and Methods

### Reagents

Apilimod was synthesized by Synta Pharmaceuticals Corp. (Lexington, MA).

### Clinical and Non-clinical (Normal Control) Study Design

Patients with moderate to severe chronic plaque psoriasis were enrolled in the multi-center, open-label, multiple oral dose outpatient study of apilimod (Protocol No. 5326–05). Eligible patients included men and women, age 18–70 years old, who had stable chronic plaque psoriasis (diagnosed at least 6 months before screening) and who had psoriasis affecting ≥ 10% of body surface area. Patients received apilimod 21mg BID, 35mg QD, 35mg BID, or 70mg QD orally for 12 weeks. Skin punch biopsies were collected for analysis of immunohistochemistry and RT-PCR at baseline (both non-lesional and lesional skin) and week 2, 6, and 12 (lesional). Whole blood was collected in sodium-heparin tubes for *ex vivo* cytokine production immediately before the administration and 2h post-dose on day 1, pre-dose on week 2 and 12. Whole blood was also collected in EDTA tubes for cell phenotype analysis pre-dose at baseline (week 0), week 2 and 12. Sixty-five patients were enrolled, and 45 (69.2%) patients completed the study. The per-protocol population consisted of 51 patients: 16, 11, 12, and 12 in the 35mg QD, 21mg BID, 35mg BID, and 70mg QD dose groups, respectively. The biopsy specimens were divided into two samples; one sample was frozen OCT media for immunohistochemistry, and the other was snap frozen in liquid nitrogen for RT-PCR. Both samples were stored at -70°C. Histological improvement of epidermal hyperplasia was defined by epidermal thickness, rete elongation, differentiation status of keratinocytes, and expression of K16 [negative in normal epidermis [39]]. Clinical response was assessed using the Psoriasis Area and Severity Index (PASI) and the static Physician’s Global Assessment (sPGA).

As an untreated control for the evaluation of biologic response, whole blood was collected independently from 18 normal volunteers at Synta Pharmaceuticals for analysis of *ex vivo* cytokine production and cell phenotype with the schedule matched with the blood collection from patients.

Both studies in patients and normal volunteers were conducted in compliance with the Declaration of Helsinki Protocols. Prior to study initiation, Schulman Associates Institutional Review Board approval (21 Code of Federal Regulations, Part 56; Cincinnati OH) was obtained for the original protocol and amendments. All patients provided written informed consent.

### Immunohistochemistry

Tissue sections were stained with hematoxylin (Fisher) and eosin (Shandon, Pittsburgh) and purified mouse anti-human mAbs to K16 (Sigma), CD3 (Becton Dickinson), CD11c (BD Pharmingen) and IL-12/IL-23p40 (R&D Systems). Biotin-labeled horse anti-mouse antibody (Vector Laboratories) was detected with avidin–biotin complex (Vector Laboratories) and developed with chromogen 3-amino-9-ethylcarbazole (Sigma Aldrich). Epidermal thickness measures were computed by using National Institutes of Health software (NIH IMAGE 6.1), and positive cells were counted using computer-assisted image analysis, as previously described [40].

### Analysis of Tissue mRNA Gene Expression

RNA was extracted from tissues frozen in liquid nitrogen by using the RNeasy Mini Kit (Qiagen, Valencia, CA). The primers and probes for TaqMan RT-PCR assays for K16, IFN-γ, TNF-α, iNOS, IL-8, IL-10, IL-17, IL-12/IL-23p40, and IL-23p19 were generated with the PRIMER EXPRESS algorithm, version 1.0. All primers and probes were purchased from Applied Biosystems. The RT-PCR was performed with EZ PCR Core Reagents (Applied Biosystems) according to the manufacturer’s directions. The samples were amplified and quantified on an Applied Biosystems PRISM 7700 by using the following thermal cycler conditions: 2 min at 50°C; 30 min at 60°C; 5 min at 95°C; and 40 cycles of 15 sec at 95°C followed by 60 sec at 60°C. The human acidic ribosomal phosphoprotein PO (hARP) gene, a housekeeping gene, was used to normalize each sample and each gene. The data were analyzed and quantified using the software provided with the Applied Biosystems PRISM 7700 (SEQUENCE DETECTION SYSTEMS, version 1.7). Skin biopsies that had degraded RNA defined by < 500 hARP or showed irregular mRNA amplification curves were excluded from the analysis [41].

### *In Vitro* and *Ex Vivo* Stimulation of whole Blood Samples for Cytokine Production

*In vitro* assay was conducted in the presence or absence of apilimod prepared in DMSO with the final DMSO concentration adjusted to 0.25% in all cultures, including the compound-free control. *Ex vivo* assay was conducted with whole blood in collection tube shipped at ambient temperature overnight from the clinical sites. Within 24h of collection, whole blood was stimulated with equal volumes of RPMI 1640 media containing a final concentration of 0.1% of *Staphylococcus aureus* Cowan I (SAC) (Calbiochem, La Jolla, CA) for 22–24h. The supernatants were harvested on the next day, and analyzed for IL-12, IL-10, and GM-CSF using Bio-Plex assays (Bio-Rad). In the *ex vivo* assay, the level of IL-12 was also confirmed using high sensitivity Quantikine ELISA Kit (R&D Systems).

### Cellular Phenotyping

The protocol included 49 three-color cell surface assays performed by microvolume laser scanning cytometry (MLSC) on the SurroScan™ system (Biomarker Discovery Sciences, PPD, formerly SurroMed) [42], [43]. Briefly, monoclonal antigen-specific antibodies were purchased from various commercial vendors, coupled to red emitting fluorophores and developed into cellular assays. Phenotypes were determined based on the presence of the following antigens: CD4^+^, T cells; CD8^+^, T cells; CD19^+^/CD20^+^, B cells; CD14^+^, monocytes; CD66b^+^, eosinophils; CD16^+^, neutrophils. Staining began within 30 h of sample collection. Aliquots of whole blood or red blood cell-lysed blood from EDTA collection tubes were added to 96-well micro-titer plates containing the appropriate reagent cocktails, incubated in the dark at room temperature for 20min, diluted with an appropriate buffer and loaded into Flex32™ capillary arrays (PPD) and analyzed with SurroScan™. Images were converted to a list-mode data format with in-house software. Fluorescence intensities were compensated for spectral overlap of the dyes so values are proportional to antigen density. Standard template gates were established using FlowJo™ cytometry analysis software (Tree Star, Inc., Ashland, OR) customized for PPD Biomarker/SurroMed to enable upload of gates to our Oracle database. Gating information was applied to the scan data for each assay to generate the resulting cell count and antigen intensity data.

### Statistical Analyses

The change over time in immunochemistry values was compared between histological responders versus non-responders using a two by four mixed analysis of variance (ANOVA) model (Table 2). These time changes were compared across the four dose groups using a four by four mixed ANOVA. Given the wide variability in the data and the small sample sizes, it was determined that a non-parametric approach was most suitable to make these same comparisons for the RT-PCR data. To do this, each variable was expressed as the difference from baseline as a percent of baseline. These percent changes were then compared across the four treatment groups using the Kruskal-Wallis one-way analysis of variance by ranks. Comparison of these percent changes between responders and non-responders was made using the Wilcoxon Ranks Sums Test.

Comparison of the absolute changes in cytokines from baseline to 2h post-dose within the 70mg QD group was made using the Wilcoxon Signed Ranks test (Fig. 2). Changes in the gene expression levels from baseline to week 2 in the 70mg QD group were also compared using the Wilcoxon Signed Ranks test (Fig. 5). This test was also used to compare changes in the gene expression levels from baseline to week 12 for both responders and non-responders (Fig. 6).

Statistical analyses for the cellular phenotyping considered the following comparisons: 1) between psoriasis subjects and healthy controls, 2) within group before and after drug treatment, and 3) between histological responders and non-responders. For all between group statistics, we applied a univariate mean comparison test that was either parametric or non-parametric depending on the normality of the data. Goodness-of-fit statistics (Shapiro-Wilk) and tests of skewness and kurtosis are performed to assess normality. If the data were approximately normally distributed in both groups, the parametric statistic was used (t-test); if not, the nonparametric rank test (Wilcoxon rank sum test) was applied. All tests of hypotheses were two-sided. Paired two-group comparison for normal controls and psoriasis subjects was designed to identify differences associated with the drug, independent of clinical outcome. The control comparisons were expected to not show differences and provide a check on the level of false positives.
